# An ILP solution for the gene duplication problem

**DOI:** 10.1186/1471-2105-12-S1-S14

**Published:** 2011-02-15

**Authors:** Wen-Chieh Chang, Gordon J Burleigh, David F Fernández-Baca, Oliver Eulenstein

**Affiliations:** 1 Department of Computer Science, Iowa State University, Ames, 50011, USA; 2 Department of Biology, University of Florida, Gainesville, 32611, USA

## Abstract

**Background:**

The gene duplication (GD) problem seeks a species tree that implies the fewest gene duplication events across a given collection of gene trees. Solving this problem makes it possible to use large gene families with complex histories of duplication and loss to infer phylogenetic trees. However, the GD problem is NP-hard, and therefore, most analyses use heuristics that lack any performance guarantee.

**Results:**

We describe the first integer linear programming (ILP) formulation to solve instances of the gene duplication problem exactly. With simulations, we demonstrate that the ILP solution can solve problem instances with up to 14 taxa. Furthermore, we apply the new ILP solution to solve the gene duplication problem for the seed plant phylogeny using a 12-taxon, 6, 084-gene data set. The unique, optimal solution, which places Gnetales sister to the conifers, represents a new, large-scale genomic perspective on one of the most puzzling questions in plant systematics.

**Conclusions:**

Although the GD problem is NP-hard, our novel ILP solution for it can solve instances with data sets consisting of as many as 14 taxa and 1, 000 genes in a few hours. These are the largest instances that have been solved to optimally to date. Thus, this work can provide large-scale genomic perspectives on phylogenetic questions that previously could only be addressed by heuristic estimates.

## Background

With recent advances in DNA sequencing technology, there is much interest in using genomic data sets to infer phylogenetic trees. However, evolutionary events such as gene duplication and loss, incomplete lineage sorting (deep coalescence), and lateral gene transfer can produce discordance between gene trees and the phylogeny of the species in which the genes evolve (e.g., [1]). The gene tree parsimony (GTP) problem [1-4] provides a direct approach to infer a species phylogeny from discordant gene trees. Given a collection of gene trees, this problem seeks a species tree that implies the minimum reconciliation cost, i.e., the fewest number of evolutionary events that can explain discordance in the gene phylogenies.

One of the most widely studied variants of the GTP problems is the gene duplication (GD) problem, in which the reconciliation cost is based on gene duplication events. The GD problem is *W*[2]-hard when parameterized by the number of gene duplications events and hard to approximate better than a logarithmic factor [5]. One way to cope with this intractability in practice is using heuristics [6,7]. Although these heuristics do not guarantee optimal solutions or any non-trivial theoretical bound, in many cases they appear to have produced credible estimates [8-11]. However, the lack of performance guarantees makes the pursuit of exact solutions for the GD problem desirable.

Exact solutions can be found by exhaustive search for every NP-complete problem, but run times typically become prohibitively large for even rather small sized instances. However, exact algorithms that are substantially faster than exhaustive search have been progressively developed (e.g. [12,13]). Unfortunately, little work has focused on such algorithms for the GD problem [14]. Here, we describe an ILP formulation solving the GD problem exactly and demonstrate its performance on both simulated and empirical data sets.

### Related work

Exact solutions to the GD problem were obtained by exhaustively searching all possible species trees in data sets with up to 8 taxa [15,16]. More recently, a branch-and-bound algorithm to identify exact solutions for the GD problem was introduced [14]. This algorithm was applied to a data-set consisting of 1, 111 gene trees with 29-taxa, but it did not run to completion. However, the branch-and-bound algorithm was able to solve this instance on reduced search spaces that resulted from providing some of the relationships in the species tree. Although constraining the search space for a species tree can help solving difficult instances of the GD problem, there are no theoretical guarantees to support this approach.

ILP formulations have provided an effective strategy to solve moderately sized instances of several NP-hard phylogenetic problems (e.g. [17-22]). Most similar to the GD problem, ILP formulations have been introduced for the deep coalescence problem, the variant of the GTP problem in which the reconciliation cost is based on the deep coalescence events [23]. These formulations solved instances with up to 8 taxa. However, perhaps due to the difficulty of directly expressing gene duplications in terms of linear equations, there have been no ILP formulations for the DP problem.

### Our contributions

We introduce a novel approach to solve the GD problem exactly by describing the first ILP formulation for this problem. This solution is made possible by revealing an alternate description of the GD problem, called the triple inconsistency problem, which expresses gene duplications in terms of rooted triples. Rooted triples are rooted full binary trees with three leaves, and are the smallest unit of phylogenetic information. They, together with an equivalent presentation of species trees through cluster hierarchies, provide the fundamental elements of our ILP solution.

We demonstrate that our ILP formulation can solve non-trivial instances with up to 14 taxa and 1,000 gene trees. This greatly improves upon the largest (unconstrained) instances of the GD problem that have been solved exactly to date. Finally, we use the ILP formulation to address the relationships among the major seed plant lineages.Our ILP formulation was able to solve the GD problem exactly for a 12-taxon data set using 6,084 gene trees.

## Methods

### Preliminaries

#### Basic definitions

A *rooted tree T* is a connected and acyclic graph consisting of a vertex set *V*(*T*)*,* an edge set *E*(*T*)*,* and that has exactly one distinguished vertex called *root,* which we denote by Rt(*T*). Let *T* be a rooted tree. We define ≤*_T_* to be the partial order on *V(T),* where *u* ≤*_T_ v* if *v* is a vertex on the path between Rt(*T*) and *u.* Moreover, we write *u <>_T_ v* if neither *u* ≤*_T_ v* nor *v* ≤*_T_ u* is true. The set of minima under ≤*_T_* is denoted by L(*T*) and its elements are called *leaves.* We call *u* a *child* of *v* if *u* ≤ *v* and {*u,v*} ∈ *V*(*E*)*.* The set of all children of *v* is denoted by Ch*_T_*(*v*). For a vertex *v* ∈ *V*(*T*) we denote by *T*(*v*) the subtree of *T* that consists of all vertices *u* ≤*_T_ v.* The *least common ancestor* of a non-empty subset *X* ⊆ *V*(*T*)*,* denoted as LCA*_T_*(*X*), is the unique smallest upper bound of *X* under ≤*_T_. T* is called *full binary* if every vertex has either two or zero children. Throughout this work, the term tree refers to a full and rooted binary tree.

#### Gene duplication (GD) problem

The terms *species tree* and *gene tree* refer to trees that represent the evolutionary history of a gene family or species respectively.

To compare a gene tree with a species tree, a mapping from each gene in the gene tree to the most recent species in the species tree that could have contained the gene is required.

**Definition 1** (Mapping). *Let G be a gene tree and S a species tree. A* leaf-mapping *from G to S is a function****L****_G,S_* : L(*G*) → *L*(*S*)*. The* extension ***M****_G_*_,_*_S_*: *V*(*G*) → *V*(*S*) *of the leaf-mapping ****L****_G_*_,_*_S_ is the mapping defined by**** M****_G,S_*(*u*) := LCA*_S_*(**L***_G,S_*(*G*(*u*))*.*

To simplify the exposition we shall assume that leaf-mappings are injections, and w.l.o.g. we identify the genes with the species from which they were sampled. After describing our ILP solution for identity leaf-mappings, we extend this formulation to cover non-injective leaf-mappings.

**Definition 2** (Comparable). *Let S be a species tree. A gene tree G is* comparable *to S, denoted by G* ⊢ *S, if****L****_G,S_ exists. A set of gene trees is* comparable *to S, denoted by****G*** ⊢ *S, if G* ⊢ *S for each gene tree G* ∈ ***G****.*

We shall adopt the following notation: we use *S* for a species tree, ***G*** for a set of gene trees that is comparable to *S,* and *G* for an gene tree in ***G****.*

**Definition 3** (Duplication). *A node g* ∈ *V*(*G*) *is a* duplication *(w.r.t. S) if****M****_G_*_,_*_S_*(*g*) ∈ ***M****_G_*_,_*_S_*(Ch*_G_*(*g*)).

For consistency we follow the common practice to call what is stated above a definition, even though it is actually a theorem [24] that follows from the gene duplication model [2].

**Definition 4** (Duplication cost). *We define the following duplication costs:*

*1.* Dup(*G*, *S*) := *|*{*g* ∈ *V*(*G*): *g is a duplication*}| *is the* duplication cost *from G to S.*

*2. *Dup(***G****, S*) := ∑_G∈_***_G_*** Dup(*G*, *S*) *is the* duplication cost *from **G** to S.*

*3.* Dup(***G***) := min***_G_***_⊢_*_T_* Dup(***G***, *T) is the* duplication cost *of****G***.

**Problem** 1 (Gene-Duplication (GD)).

Instance: A set of gene trees **G**.

*Find: The duplication cost* Dup(***G***), and a species tree S* such that Dup(***G***, S*) *= Dup*(***G***)*.*

### The Triple-Inconsistency problem and its equivalence to the GD problem

A *rooted triple* is a tree with three leaves. The rooted triple with leaves *x*, *y*, and *z* is denoted *xy*|*z* if the path between *x* and *y* does not intersect with the path between *z* and the root. A rooted triple is *displayed* by a tree *T* if LCA*_T_*(*x*, *y*) ≤_*T*_ LCA_*T*_(*x*, *z*) (= LCA*_T_*(*y*, *z*))*.* The set of rooted triples *xy*|*z* displayed by tree *T* that are rooted at vertex *u* ∈ *V*(*T*)*,* (i.e., *u = LCA_T_*({*x*, *y*, *z*})) is denoted by Trip*_T_*(*v*), and the set of all triples displayed by *T* is denoted by Trip(*T*).

**Definition 5** (T(riple)-inconsistency). *A rooted triple t* ∈ Trip(*G*) *is said to be* inconsistent *with S if t* ∉ Trip(*S*). *A vertex v* ∈ *V*(*G*) *is called* t(riple)-inconsistent *with S if there is a rooted triple in* Trip_G_(*v*) *that is inconsistent with S.*

**Definition 6** (T-inconsistency cost). *We define the following t-inconsistency costs:*

*1.* Tin(*G,S*) :*= |*{*v* ∈ *V*(*G*)*: v is t-inconsistent with S*}| *is the* t-inconsistency cost *from G to S.*

*2.* Tin(***G***, *S*) :*= ∑_G_*_∈_***_G_*** Tin(G, *S*) *is the* t-inconsistency cost *from **G** to S.*

*3.* Tin(***G***) := min**_G_**_⊢T_ Tin(***G***,*T*) *is the* t-inconsistency cost *of **G**.*

**Problem** 2 (T(riple)-inconsistency).

Instance: A set of gene trees **G**.

*Find: The t-inconsistency cost Tin*(***G***), and species tree S* such that Tin(***G***, S*) = Tin(***G***)*.*

**Theorem** 1 (Equivalence between duplication and t-inconsistency). *Let u* ∈ (*G*)*. Then u is a duplication w.r.t. S if and only if u is t-inconsistent with S.*

*Proof.* Let *x* := ***M****_G,S_*(*u*)*.*

Suppose *u* is a not a duplication. Let *ab|c* ∈ Trip_G_(u). We will show that *ab|c* ∈ Trip(S). By the definition of *ab|c* ∈ Trip_G_(u) we know that LCA_G_({α, *b,c*}) = *u*, and together with our assumption that *G* is fully binary it follows that *u* has two children *v* and *w,* where w.l.o.g. *a, b* ∈ L(*G*(*v*)) and c ∈ L(*G*(*w*))*.* Let *v*′ := ***M****_G,S_*(*v*) and *w*′ := ***M****_G,S_*(*W*)*.* From *a, b* ∈ L(*G*(*v*)) and *c* ∈ L(*G*(*w*)) follows that *a, b* ∈ L(*S*(*v*′)) and *c* ∈ L(*S*(*w*′)) respectively. Now, since *u* is not a duplication we have *v*′ *<>_S_ w*′*.* Otherwise, we would have *w*′ ≤*_S_ v*′ or *v*′ ≤*_S_ w*′ from which *x = v*′ or *x = w*′ would follow respectively; contradicting that *v* is not a duplication. Hence, from *v*′ *<>_S_ w*′ and *a, b* ∈ L(*S*(*v*′)) and c ∈ L(*S*(*w*′)) follows that *ab|c* ∈ Trip(*S*).

Suppose *u* is a duplication, and thus we have *x* = ***M****_G,S_*(*v*) for a child *v* ∈ Ch(*u*)*.* So *u* is not a leaf in G, and since G is fully binary it follows that there are two distinct vertices *a, b* ∈ L(*G*(*u*)) such that LCA*_S_*({*a,b*}) *= x.* Therefore, *x* has two children *y* and *z* such that *a* ≤*_S_ y* and *b* ≤*_S_ z.* Now we distinguish different cases for the vertices *a* and *b* based on their possible order relation to the children of *u.* Since *G* is fully binary and *v* is a child of *u*, there exists a child *w* ∈ Ch(*u*) where *w* ≠ *v.* Now, we have the following cases.

Case 1: *a* ≤*_G_ v, b* ≤*_G_ v*: Let *c* ≤*_G_ w.* Then *ab|c* ∈ Trip*_G_*(*u*). Further *c* ≤*_S_ y* or c ≤*_S_*z and with *a* ≤*_S_y* and *b* ≤*_S_ z,* it follows that either *ac|b* ∈ Trip_s_(x) or *bc|a* ∈ Trip*_S_*(*x*). Hence, *u* is t-inconsistent as desired.

Case 2: *a* ≤*_G_ v, b* ≤*_G_ w:* We know that *x* has two children *y* and *z* and that ***M****_G,S_*(*v*) *= x.* Therefore there exist *c* ≤*_S_ y* and *d* ≤*_S_ z* such that LCA_s_(c, *d*) = ***M***(*v*) where *c,d* ∈ L(*G*(*v*))*.* From the order relations *a* ≤*_S_ y, d* ≤*_S_ z* and *d* ≤*_G_ v, b* ≤*_G_ w* and *a* ≤*_G_ v, b* ≤*_G_ w,* it follows that *a, b* and *d* are pairwise different. Therefore the rooted triples *ad|b* ∈ Trip*_G_*(u) and *bd|a* ∈ Trip*_S_*(*x*) are well defined, from which follows that the vertex *u* is t-inconsistent.

Case 3: *a* ≤*_G_ W, b* ≤*_G_ w* or *b* ≤*_G_ v, a* ≤*_G_ w*: Similarly to the previous cases it follows that *u* is t-inconsistent

From Theorem 1, the next corollary follows.

**Corollary 1** (Equivalence between the GD problem and the T-Inconsistency problem). *The t-inconsistency problem is a mathematical equivalent formulation of the duplication problem (i.e.* Dup(*G,S*) = Tin(*G,S*)*)*.

### An ILP solution for the T-Inconsistency problem

Table 1 lists the variables used, and their meaning. To explain our ILP solution, we first formulate all possible candidate trees in the solution space of the t-inconsistency problem. Next we formulate the t-inconsistency objective to identify an optimal t-inconsistency cost and an optimal candidate tree.

**Table 1:** 

Notation	Definition
*M*(*i*, *j*)	Taxon-cluster representation of (the) species tree: *M*(*i*, *j*) = 1 iff taxon *i* is in the cluster *j.* Additional constraints on *M* require the cluster set to form a binary hierarchy (tree).
*C*(*p*, *q*, *xy*)	Compatibility: *C*(*p*, *q*, *xy*) = 1 exactly if the cluster pair (*p*, *q*) has the gamete *xy* ∈ {01, 10, 11}.
*T*(*a*, *b*, *c*, *xyz*)	Rooted triple: *T*(*a*, *b*, *c*, *xyz*) = 1 exactly if the rooted triple with leaf set {*a*, *b*, *c*} and topology *xyz* is displayed in *M.* Topologies for *xyz* are 011, 101, and 110 and refer to the rooted triples *bc|a*, *ac|b* and *ab|c* respectively.
*D*(*g*)	t-inconsistency: *D*(*g*) = 1 if the gene vertex *g* is t-inconsistent w.r.t. a tree represented by matrix *M.*

Notation used in our ILP solution.

Let *X* := ∪*_G_*_∈_***_G_*** L(*G*) be the taxon set, *n* := |*X*|, m := |∪*_G_*_∈_***_G_***Trip(*G*)|, and *k* :*= |****G****|.* It follows that Σ*_G_*_∈_***_G_****|G| = O*(*kn*)*.*

#### Formulating candidate species trees in terms of cluster hierarchies

Here we formulate constraints that describe all species trees that are possible candidates for solving the t-inconsistency problem, that is, all trees to which the given gene tree set ***G*** is compatible. Based on our assumption that the leaf label function is the identity function, these are all trees with the leaf set *X.* Our ILP formulation is based on an alternative way of describing trees by specifying their clusters through a hierarchy of subsets of *X.*

**Definition 7** (Clusters). *Let T be a tree. For each vertex v* ∈ *V*(*T*) *we define the* cluster *at v as* {*u* ∈ L(*T*) : *u* ≤*_T_ v*}*, i.e.,* L(*T*(*u*))*. We shall denote the set of all clusters of T by****H***(*T*)*.*

**Definition 8** ((Full) Binary hierarchy). *Let F be a finite set. We call a set **H** of non-empty subsets of F a* (full) binary hierarchy *on F if the following properties are satisfied:*

*1. Trivial set property: F* ∈ ***H** and* {*v*} ∈ ***H** for each v* ∈ *F*

*2. Compatibility property: every pair of sets A and B in **H** is* compatible; *that is A ∩ B* ∈ {*A, B,* Ø}.

*3. Cardinality property*: *|****H****|=* 2|*F*| –1

A *hierarchy* is defined as a binary hierarchy without requiring the cardinality property. There is a well-known and fundamental equivalence between hierarchies and trees that are not necessarily binary (e.g. [25]). The next result follows from this equivalence and the fact that a binary tree over *l* leaves has exactly *2l* – 1 clusters.

**Theorem** 2 (Equivalence between binary hierarchies and binary trees). *Let **H** be a set of non-empty subsets of a set F. Then there is a binary tree T such that****H*** = ***H***(*F*) *if and only if **H** is a binary hierarchy on F.*

Since trees and binary hierarchies are equivalent, we use these terms interchangeably from now on. Now we formulate constraints that describe the hierarchies on *X* using the binary matrix presentation.

Binary matrix**.** We describe 2*n* – 1 subsets of a hierarchy on *X* using a binary matrix *M* with a row for each species in *X* and *2n* – 1 columns, where each column *p* represents the set {*a* ∈ *X*: *M*(*a,p*) = 1}.

**Excluding sets satisfying the trivial set property.** We consider only the *n* – *2* non-trivial sets that can be part of a binary hierarchy on *X.* To do this, we add the following constraints that allow only non-trivial sets. For each column *p* of *M,* we require

2 ≤ Σ*_a_*_∈_*_X_M*(*a*, *p*) ≤ (*n* – 1).

**Uniqueness.** To ensure that a set of subsets is uniquely represented by the columns of *M,* we enforce a linear order of a binary interpretation of these columns. Suppose that *X =* {*a*_1_,…,*a_n_*} are the rows of *M,* then this order is achieved by adding the following (*n* – 3) constraints that apply to all pairs of adjacent columns *p* and *q* in *M*.

**Compatibility.** Incompatibility can be tested directly by using the three-gamete condition (e.g., [26]). An incompatibility occurs for two columns *p* and *q* in *M* if and only if there exist three rows *a, b* and c in *M* that contain the *gametes* (0,1), (1,0), and (1,1) in *p* and *q* respectively (i.e. (*M*(*a,p*),*M*(*a*, *q*)) = (0,1), (*M*(*b*, *p*),*M*(*b,q*)) = (1,0), and (*M*(*c*, *p*),*M*(*c*, *q*)) = (1,1)). To identify if a certain gamete (*x*, *y*) ∈ {(0,1), (1,0), (1,1)} exists for *p* and *q,* we define a set of binary variables *C*(*p*, *q*, *xy*) under the following constraints over all rows *a* in *M.*

*C*(*p, q,* 01) ≥ –*M*(*a*, *p*) *+ M*(*a*, *q*),

*C*(*p, q,* 10) ≥ *M*(*a, p*) – *M*(*a, q*),

*C*(*p, q,* 11) ≥ *M*(*a, p*) *+ M*(*a, q*) – 1.

These constraints capture that *C*(*p*, *q*, *xy*) = 1 as long as *M*(*a, p*) *= x* and *M*(*a, q*) *= y* is satisfied for a gamete (*x, y*) in a certain row *a* in *M.* However, the reverse condition does not necessarily hold true without adding further constraints. To guarantee that clusters *p, q* are compatible, we require the following constraints

*C*(*p, q,* 01) + *C*(*p, q,* 11) *+ C*(*p, q,* 10) = 2.

**Number of variables and constraints.** There are *O*(*n^2^*) variables for the matrix *M,* and *O*(*n*^2^) variables of the type *C*(*p*, *q*, *xy*)*. O*(*n*) constraints are needed to exclude trivial sets and to guarantee uniqueness, and *O*(*n^3^*) constraints guarantee compatibility. In summary, there are *O*(*n*^2^*)* variables and *O*(*n*^3^) constraints to describe all candidates for the species tree.

**Formulating the T-lnconsistency problem.** To formulate the t-inconsistency problem, we first describe variables *T*(*a, b, c, xyz)* that detect whether a rooted triple is displayed by the tree presented by *M.* Then we describe variables *D*(*g*) that detect if *g* is t-inconsistent by using the variables *T*(*a, b, c, xyz*)*.* Finally, we formulate the objective of the t-inconsistency problem based on the variables *D*(*g*)*.*

**Variables *T*(*a, b, c, xyz*)*.*** We describe the binary variables *T*(*a, b, c, xyz*) that are 1 exactly if a rooted triple over the leaf set {*a, b, c*} with topology (*x*, *y*, *z*) ∈ {(0,1,1), (1,0,1), (1,1,0)} is displayed by the tree that is presented by *M.* The parameters *a, b, c* are rows in *M,* and the settings 011, 101, and 110 of (*x*, *y*, *z*) refer to the rooted triples *bc|a, ac|b* and *ab|c* respectively. For each column *p* in *M,* we introduce the following constraints.

*T*(*a, b, c,* 011) ≥ –*M*(*a, p*) *+ M*(*b, p*) *+ M*(*c, p*) – 1 ;

*T*(*a, b, c,* 101) ≥ *M*(*a, p*) – *M*(*b, p*) *+ M*(*c, p*) – 1 ;

*T*(*a, b, c,* 110) ≥ *M*(*a, p*) *+ M*(*b, p*) *- M*(*c, p*) – 1 ;

*T*(*a, b, c,* 011) + *T*(*a, b, c,* 101) + *T*(*a, b, c,* 110) = 1 ,

since a rooted triple is uniquely resolved in a tree.

Variables *T*(*a, b, c,* 011), *T*(*a, b, c,* 101), and *T*(*a, b, c,* 110) are constructed for every triple {*a, b, c*} for which a rooted triple is displayed by a gene tree in *.* Thus, there are *O*(*m*) variables of this type. For each variable we have *O*(*n*) constraints, which results in *O*(*nm*) constraints overall.

**Variables *D*(*g*)*.*** We express the t-inconsistency of each vertex *g* ∈ *V*(*G*) where *G* ∈ ***G*** by the binary variable *D*(*g*)*.* The variable is 1 if *g* is t-inconsistent with the tree described by matrix *M,* given the following constraints

*D*(*g*) ≥ 1 – *T*(*a, b, c, xyz*) *,*

where the rooted triple over the leaf set {*a, b, c*} and topology *xyz* is an element in Trip*_G_*(*g*).

Variables *D*(*g*) are constructed for each internal vertex of a gene tree in ***G****,* which results in *O*(*kn*) variables. Intuitively, a constraint is constructed for each rooted triple that is displayed by a gene tree in *G,* which yields *O*(*km*) constraints. However, the following observation reduces the number of such constraints to *O*(*kn*^2^)*.*

Let *u* ∈ *V*(*G*) such that Trip_G_(u) ≠ Ø, {*v,w*} = Ch(u), *a* ∈ L(*G*(*u*)) and *b* ∈ L(*G*(*v*))*.* A rooted triple *xy|z* is in Trip_G_(u) if and only if all *ax|b, ay|b,* and *bz|a* are in Trip_G_(u). Therefore, instead of enumerating all rooted triples in Trip_G_(u) (which sums up to *O*(*m*) in each gene tree *G*), we only need to enumerate a number of *O*(*n*) rooted triples to represent Trip*_G_*(u) while detecting if *u* is t-inconsistent (hence *O*(*kn^2^*) constraints over all).

**T-lnconsistency objective.** This objective is expressed by the following expression.

min Σ_g∈_*_V_*_(_***_G_***_)_*D*(*g*).

Once the optimal objective cost is found, a unique tree corresponding to the cost can be constructed from *M.* It is worth noting that an instance of unique optimal tree does not ensure an unique optimal solution to the corresponding ILP due to relaxed constraints for variables *C.* Although this can be addressed by adding additional constraints, the correctness of the objective value and the resulting tree is not affected.

**Number of variables and constraints.** In summary, there are *O*(*n*^2^ + m + *kn*) variables, and the number of constraints is *O*(*n*^3^*+ mn + kn*^2^)*.*

#### Handling non-injective leaf mappings

A leaf mapping ***L****_G,S_* is non-injective if and only if there is a vertex *u* ∈ *V*(*G*) with distinct children *v* and *w* such that ***L****_G,S_*(L(*G*(*v*)))Ø***L****_G,S_*(L(*G*(*w*))) ≠ Ø; and if the latter holds true, it follows that *u* is a duplication. Therefore, it can be determined if *u* is a gene-duplication regardless of the topology of *S.* By pre-processing all such determined duplication vertices, the leaf-mapping over the remaining internal vertices of G can be made injective. Hence, the existing ILP formulation solves input gene trees with non-injective leaf mappings. Since the input gene tree size can be arbitrary, under the non-injective leaf mapping assumption, the ILP formulation has *O*(*n*^2^*+ m + l*) variables and *O*(*n*^3^*+ mn + In*) constraints where Σ*_G_*_∈_***_G_****|G| = l.*

#### Generating optimal species trees

The species tree corresponding to a feasible solution of an ILP instance can be constructed in *O*(*n*^2^) time [27]. Furthermore, a gene node g is identified as a duplication if and only if *D*(*g*) *= l.*

#### Implementation

We implemented an ILP generator in Python that, given a set of gene trees, outputs the ILP described in the preceding section. We tested our formulation with both simulated and empirical gene tree data sets (described below). All analyses were on a GNU/Linux based PC with an Intel Core2 Quad 2.4 GHz CPU. We choose Gurobi 2.0 [28] to solve the ILP directly and CPLEX 12.1 [29] to enumerate optimal solutions when necessary.

#### Simulation experiments

We first evaluated the performance of our ILP solution with simulated gene tree data sets. Our simulation protocol included the following steps: (1) a species tree *S* of *n* taxa was randomly generated as the template of a gene tree; (2) a depth-first-search walk starting from Rt(S) simulated at most one evolutionary event at each vertex based on given probabilities for each event. These events could be a duplication (duplicating the whole current subtree) or a loss (cutting the current subtree). If there is neither a duplication nor a loss, the process proceeds to the next vertex. We used the same species tree to generate *k* gene trees.

In our simulation experiments, we used a duplication rate of 0.25 duplications per gene at each spe-ciation vertex and a loss rate of 0.3. These events produced a similar tree size distribution and optimal duplication cost to the gene trees used by Sanderson and McMahon [16]. We varied the number of taxa in the species tree from 6 to 14 and the number of input gene trees from 10 to 1000. We performed 10 simulation replicates for each different combination of species and gene tree number. For each simulated data set, we also compared the ILP score to results from DupTree [7], a fast hill-climbing heuristic implementation for the problem, to determine if the heuristic finds the optimal solution.

#### Seed plant analysis

Next, we tested the ability of the ILP formulation to solve the seed plant phylogeny problem using a large-scale genomic data set. First, to build the gene trees, amino acid alignments for gene families were selected from Phytome v. 2, an online comparative genomics database based on publicly available sequence data from 136 plant species [30]. To ensure positional homology throughout the alignments, columns and sequences of questionable certainty were masked using default settings of the program REAP [30,31].We sampled sequences from the nine gymnosperm taxa represented in Phytome with the most data, including cycad taxon *Cycas rumphii,* Gnetales taxa *Gnetum gnemon* and *Welwitschia mirabilis*, and, from the conifers, *Cryptomeria japonica* from Cupres-saceae, and *Pseudotsuga menziesii*, *Picea glauca*, *Picea sitchensis*, *Pinus pinaster*, and *Pinus taeda* from Pinaceae. We also sampled sequences from two representative angiosperm taxa, *Arabidopsis thaliana* and *Oryza sativa*, and the non-seed plant, *Physcomitrella patens*.

We selected all the 6,084 masked amino acid alignments from gene families in Phytome that had at least 4 sequences and had sequences from at least 3 of the selected taxa. All species were found in at least 376 gene families. To build the gene trees, we performed ML phylogenetic analyses on each of the gene alignments using RAxML-VI-HPC version 2.2.3 [32]. The ML analyses used the JTT amino acid substitution model [33] with rate variation among sites (the “PROTMIX” model; see [32]). The trees were then rooted using mid-point rooting, as implemented in the Phylip program retree [34]. We applied the ILP formulation to solve the GD problem using all 6, 084 gene trees.

## Results and discussion

### Simulations

In the simulation experiments, the size of the species tree has a major impact on running time (Table 2), but we were able to find exact solutions for the GD problem for data sets with up to 14 taxa (Table 2). On average, the 14-taxon data sets took less than 2 hours. There is no clear relationship between the number of gene trees and the time it takes to solve the GD problem (Table 2). Although the data sets with 1000 gene trees took, on average, longer to solve than data sets with fewer gene trees, in some cases with fewer gene trees (specifically, 10 gene trees) it is difficult to determine an optimal solution when the optimal species tree is not unique. In comparison, the heuristic approach used in Dup-Tree found an optimal solution in almost all of the simulated data sets under only a few seconds. However, DupTree reported suboptimal trees on some data sets with as few as 10 taxa and 10 gene trees.

**Table 2:** 

	*n* = 6		*n* = 8		*n* = 10		*n* = 12		*n* = 14	
*k*	time	Dup	time	Dup	time	Dup	time	Dup	time	Dup

10	0.06	34.80	0.34	49.70	22.98	60.10	200.53	68.80	12597.21	78.40
50	0.03	189.50	1.26	265.00	8.74	280.00	159.26	346.40	2953.62	393.10
100	0.06	382.80	0.63	523.30	9.64	598.50	117.38	701.60	2191.65	825.70
200	0.05	788.20	0.54	994.90	11.03	1217.30	168.85	1372.50	2709.91	1627.70
500	0.25	1910.30	0.79	2458.60	13.92	2987.00	220.17	3678.80	4270.05	4001.70
1000	0.57	3842.60	0.96	5283.10	23.54	6140.90	330.34	7026.40	5014.61	8258.80

ILP running time and the optimal duplication cost using *k* simulated gene trees of n taxa as inputs. At each configuration, the result is the average of 10 trials. The running time is measured in seconds.

### Seed plant analysis

The relationships among the major lineages of seed plants has long been a major question in plant systematics, especially with regard to the position of Gnetales, a clade of three genera (Gnetum, Ephedra, and Welwitschia) that lack obvious morphological links to other extant seed plants (e.g.,, [35,36,39]). Cladistic analyses of morphological characters generally have placed Gnetales sister to the angiosperms, or flowering plants [36,40-44]; however, early analyses of molecular characters rarely supported this placement [35,37,39,45]. Most recently, maximum likelihood (ML) and maximum parsimony (MP) analysis of 15-17 plastid loci placed Gnetales sister to the other seed plants [46]. However, a loss of plastid *ndh* genes appears to link Gne-tales with Pinaceae [47]. An MP analysis of EST sequences from 43 nuclear genes similarly linked Gnetales with the conifers [48]. Yet later MP and ML analyses of EST sequences from over 1,200 nuclear loci placed Gnetales sister to the other gym-nosperms [49]. All of these molecular analyses of the seed plant phylogeny have been limited to puta-tively orthologous genes. However, the GD problem provides a way to incorporate large gene families into the phylogenetic inference of seed plants.

**Figure 1:**
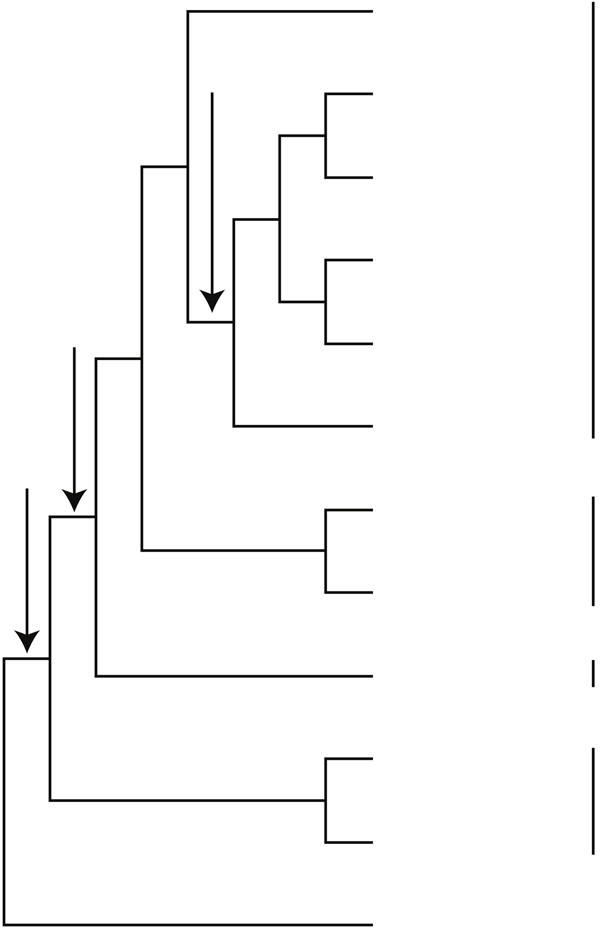
**The optimal seed plant phylogeny**. The unique optimal seed plant phylogeny based on 12 taxa and 6,084 genes under the GD model.

Our implementation of the ILP formulation finished running the data set in approximately two minutes. We identified a unique optimal solution with 47, 658 duplications (Figure 1).In the optimal species tree, the seed plants are split into angiosperm and gymnosperm clades (Figure 1). In the gymnosperm clade, Gnetales are sister to a conifer clade. With 6, 084 genes, this GTP analysis of seed plants includes by far the most genes ever used to infer the seed plant phylogeny. Our GTP analysis of this large, previously underutilized, data source provides a novel line of evidence that angiosperms and all extant gymnosperms are sister clades. Like most ML analyses of multi-locus data sets, our results show a close affinity between Gnetales and conifers (e.g., [35,37,39,50,51]); however, unlike many of these analyses, GTP does not place Gnetales sister to Pinaceae. Due to the necessarily limited taxon sampling, especially among non-Pinaceae conifers, our results regarding the placement of Gnetales are neither precise nor definitive. Still, the placement of Gnetales sister to the conifers, is an intriguing result that is consistent with some morphological characters, such as ovulate cone scales and resin canals, which appear to support conifer monophyly [36]. However, in contrast to our result, the deletion of the *ndh* genes in Gnetales and Pinaceae suggests that these clades are sister. Although the GTP results are intriguing, they should be interpreted with caution. For example, the results do not provide any measures of confidence or suggest the degree to which alternate phylogenetic hypotheses are sub-optimal. Furthermore, the gene trees were rooted using mid-point rooting, which may produce incorrect rootings when the sequences do not evolve at a constant rate of evolution [52]. Also, the taxon sampling in this analysis is limited, and the seed plant phylogeny problem can be sensitive to taxon sampling [45]. Thus, although our result provides a novel large-scale genomic perspective on the seed plant phylogeny, it is not a definitive.

## Conclusions

Our ILP formulation provides exact solutions to the largest instances of the GD problem analyzed to date. Thus, it can provide a large-scale genomic perspective on important phylogenetic questions that previously could only be addressed by heuristics. Furthermore, our simulation experiments demonstrate that these heuristic estimates can be misled with as few as 10 taxa. Even when heuristics identify an optimal solution they cannot, unlike ILP, determine if the solution is unique. In future research the ILP implementation will be useful, not only for solving empirical data sets, but for assessing the performance of different heuristics by comparing their estimates to the exact ILP solution. Ultimately, it also will be useful to expand the scale of solvable instances beyond 14 taxa. While this challenge may be addressed by improved ILP formulations, investigations into other algorithm concepts might also be effective (e.g., [14,23]).

## Authors contributions

WCC was responsible for developing the solution, running experiments, and writing of the manuscript. JGB performed the experimental evaluation and the analysis of the results, and contributed to the writing of the manuscript. OE and DFB supervised the project and contributed to the writing of the paper. All authors read and approved the final manuscript.

## Competing interests

The authors declare that they have no competing interests.

## References

[B1] Maddison WP Gene trees in species trees Syst. Biol 1997 46 523 536 10.1093/sysbio/46.3.523

[B2] Goodman M Czelusniak J Moore GW Romero-Herrera AE Matsuda G Fitting the Gene Lineage into its Species Lineage, a parsimony strategy illustrated by cladograms constructed from globin sequences Syst. Zool 1979 28 132 163 10.2307/2412519

[B3] Guigó R Muchnik I Smith TF Reconstruction of Ancient Molecular Phylogeny Mol 1996 6 2 189 213 8899723 10.1006/mpev.1996.0071

[B4] Slowinski JB Knight A Rooney AP Inferring Species Trees from Gene Trees: A Phylogenetic Analysis of the Elapidae (Serpentes) Based on the Amino Acid Sequences of Venom Proteins Mol 1997 8 3 349 362 10.1006/mpev.1997.0434 9417893

[B5] Bansal MS Shamir R A Note on the Fixed Parameter Tractability of the Gene-Duplication Problem IEEE/ACM Trans 2010 10.1109/TCBB.2010.74 20733245

[B6] Bansal MS Burleigh JG Eulenstein O Wehe A Heuristics for the Gene-Duplication Problem: A Θ(n) Speed-Up for the Local Search RECOMB, Volume 4453 of LNCS 2007 238 252

[B7] Wehe A Bansal MS Burleigh JG Eulenstein O Dup-Tree: a program for large-scale phylogenetic analyses using gene tree parsimony Bioinformatics 2008 24 13 1540 1541 10.1093/bioinformatics/btn230 18474508

[B8] Page RDM Extracting Species Trees From Complex Gene Trees: Reconciled Trees And Vertebrate Phylogeny Mol 2000 14 89 106 10.1006/mpev.1999.0676 10631044

[B9] Cotton JA Page RDM Going Nuclear: Gene Family Evolution And Vertebrate Phylogeny Reconciled Proc Biol Sci 2002 269 1555 1561 10.1098/rspb.2002.2074 12184825 PMC1691073

[B10] Martin AP Burg TM Perils of Paralogy: Using HSP70 Genes for Inferring Organismal Phylogenies Syst 2002 51 4 570 587 10.1080/10635150290069995 12228000

[B11] McGowen MR Clark C Gatesy J The Vestigial Olfactory Receptor Subgenome of Odontocete Whales: Phylogenetic Congruence between Gene-Tree Reconciliation and Supermatrix Methods Syst 2008 57 4 574 590 10.1080/10635150802304787 18686195

[B12] Applegate DL Bixby RE Chvatal V Cook WJ The Traveling Salesman Problem: A Computational Study (Princeton Series in Applied Mathematics) 2007 Princeton University Press

[B13] Woeginger GJ Exact algorithms for NP-hard problems: A survey Combinatorial OptimizationÂ–Eureka, You Shrink! 2003 2570/2003 185 207

[B14] Doyon JP Chauve C Branch-and-Bound Approach for Parsimonious Inference of a Species Tree From a Set of Gene Family Trees Tech 2010 LIRMM 10.1007/978-1-4419-7046-6_29 21431569

[B15] Burleigh JG Bansal MS Eulenstein O Vision TJ Inferring Species Trees From Gene Duplication Episodes Proc. ACM-BCB 2010 198 203

[B16] Sanderson MJ McMahon M Inferring angiosperm phylogeny from EST data with widespread gene duplication BMC Evol 2007 7 Suppl 1 S3 10.1186/1471-2148-7-S1-S3 17288576 PMC1796612

[B17] Brown DG Harrower IM Integer Programming Approaches to Haplotype Inference by Pure Parsimony IEEE/ACM Trans 2006 3 2 141 154 10.1109/TCBB.2006.24 17048400

[B18] Dong J Fernández-Baca D McMorris FR Constructing majority-rule supertrees Algorithms for Molecular Biology 2010 5 2 10.1186/1748-7188-5-2 20047658 PMC2826330

[B19] Gusfield D The Multi-State Perfect Phylogeny Problem with Missing and Removable Data: Solutions via Integer-Programming and Chordal Graph Theory RECOMB 2009 236 252 10.1089/cmb.2009.0200 20377452

[B20] Gusfield D Frid Y Brown DG Integer Programming Formulations and Computations Solving Phylogenetic and Population Genetic Problems with Missing or Genotypic Data COCOON 2007 51 64

[B21] Sridhar S Lam F Blelloch GE Ravi R Schwartz R Efficiently finding the most parsimonious phylogenetic tree via linear programming Int 2007 4463 37 48 full_text

[B22] Chimani M Rahmann S Sebastian B Exact ILP Solutions for Phylogenetic Minimum Flip Problems Proc. ACM BCB 2010 147 153

[B23] Than C Nakhleh L Species Tree Inference by Minimizing Deep Coalescences PLoS Comput 2009 5 9 e1000501 10.1371/journal.pcbi.1000501 19749978 PMC2729383

[B24] Eulenstein O Vorhersage von Genduplikationen und deren Entwicklung in der Evolution PhD dissertation 1998 University of Bonn

[B25] Semple C Steel MA Phylogenetics 2003 Oxford University Press

[B26] Gusfield D Algorithms on Strings, Trees and Sequences: Computer Science and Computational Biology 1997 Cambridge University Press

[B27] Gusfield D Efficient algorithms for inferring evolutionary trees Networks 1991 21 19 28 10.1002/net.3230210104

[B28] Gurobi Optimization, Inc Gurobi Optimization 2.0.2 2010 http://www.gurobi.com/

[B29] IBM, Inc IBM ILOG CPLEX 12.1 2009 http://www.ibm.com/software/integration/optimization/cplex/

[B30] Hartmann S Lu D Phillips J Vision TJ Phytome: a platform for plant comparative genomics Nucleic Acids Res 2006 34 Database issue D724 D730 10.1093/nar/gkj045 16381967 PMC1347408

[B31] Hartmann S Vision TJ Using ESTs for phylogenomics: Can one accurately infer a phylogenetic tree from a gappy alignment? BMC Evol 2008 8 95 10.1186/1471-2148-8-95 18366758 PMC2359737

[B32] Stamatakis A RAxML-VI-HPC: maximum likelihood-based phylogenetic analyses with thousands of taxa and mixed models Bioinformatics 2006 22 21 2688 2690 10.1093/bioinformatics/btl446 16928733

[B33] Jones DT Taylor WR Thornton JM The rapid generation of mutation data matrices from protein sequences Comput 1992 8 3 275 282 1633570 10.1093/bioinformatics/8.3.275

[B34] Felsenstein J PHYLIP (Phylogeny Inference Package) version 3.6 Distributed by the author 2005

[B35] Burleigh JG Mathews S Phylogenetic signal in nucleotide data from seed plants: Implications for resolving the seed plant tree of life Am 2004 91 10 1599 1613 10.3732/ajb.91.10.1599 21652311

[B36] Donoghue MJ Doyle JA Seed plant phylogeny: Demise of the anthophyte hypothesis? Current Biology 2000 10 3 R106 R109 10.1016/S0960-9822(00)00304-3 10679315

[B37] Magallón S Sanderson MJ Relationships among Seed Plants Inferred from Highly Conserved Genes: Sorting Conflicting Phylogenetic Signals among Ancient Lineages Am 2002 89 12 1991 2006 10.3732/ajb.89.12.1991 21665628

[B38] Mathews S Phylogenetic relationships among seed plants: Persistent questions and the limits of molecular data Am 2009 96 228 236 10.3732/ajb.0800178 21628186

[B39] Soltis DE Soltis PS Zanis MJ Phylogeny of Seed Plants Based on Evidence from Eight Genes Am 2002 89 10 1670 1681 10.3732/ajb.89.10.1670 21665594

[B40] Crane PR Phylogenetic Analysis of Seed Plants and the Origin of Angiosperms Annals of the Missouri Botanical Garden 1985 72 716 793 10.2307/2399221

[B41] Doyle JA Seed Ferns and the Origin of Angiosperms The Journal of the Torrey Botanical Society 2006 133 169 209 10.3159/1095-5674(2006)133[169:SFATOO]2.0.CO;2

[B42] Doyle JA Donoghue MJ Seed plant phylogeny and the origin of angiosperms: An experimental cladistic approach The Botanical Review 1986 52 4 321 431 10.1007/BF02861082

[B43] Hilton J Bateman RM Pteridosperms are the backbone of seed-plant phylogeny The Journal of the Torrey Botanical Society 2006 133 119 168 10.3159/1095-5674(2006)133[119:PATBOS]2.0.CO;2

[B44] Nixon KC Crepet WL Stevenson DW Friis EM A Reevaluation of Seed Plant Phylogeny Annals of the Missouri Botanical Garden 1994 81 3 484 533 10.2307/2399901

[B45] Rydin C Kallersjo M Friis EM Seed Plant Relationships and the Systematic Position of Gnetales Based on Nuclear and Chloroplast DNA: Conflicting Data, Rooting Problems, and the Monophyly of Conifers Int 2002 163 2 197 214 10.1086/338321

[B46] Rai HS Reeves PA Peakall R Olmstead RG Graham SW Inference of higher-order conifer relationships from a multi-locus plastid data set Botany 2008 86 658 669 10.1139/B08-062

[B47] Braukmann TWA Kuzmina M Stefanovic S Loss of all plastid ndh genes in Gnetales and conifers: extent and evolutionary significance for the seed plant phylogeny Current Genetics 2009 55 3 323 337 10.1007/s00294-009-0249-7 19449185

[B48] de La Torre-Bárcena JE Egan M Katari MS Brenner ED Stevenson DW Coruzzi GM DeSalle R ESTimating plant phylogeny: lessons from partitioning BMC Evol 2006 6 48 16776834 10.1186/1471-2148-6-48 PMC1564041

[B49] de La Torre-Bárcena JE Kolokotronis SO Lee EK Stevenson DW Brenner ED Katari MS Coruzzi GM DeSalle R The Impact of Outgroup Choice and Missing Data on Major Seed Plant Phylogenetics Using Genome-Wide EST Data PLoS ONE 2009 4 6 e5764 19503618 10.1371/journal.pone.0005764 PMC2685480

[B50] Burleigh JG Mathews S Assessing systematic error in the inference of seed plant phylogeny Int 2007 168 2 125 135 10.1086/509588

[B51] Wu CS Wang YN Liu SM Chaw SM Chloroplast Genome (cpDNA) of Cycas taitungensis and 56 Cp Protein-coding Genes of Gnetum parvifolium: Insights into CpDNA Evolution and Phylogeny of Extant Seed Plants Mol 2007 24 1366 1379 10.1093/molbev/msm059 17383970

[B52] Holland BR Penny D Hendy MD Outgroup Misplacement and Phylogenetic Inaccuracy under a Molecular Clock: A Simulation Study Syst 2003 52 2 229 238 10.1080/10635150390192771 12746148

